# Supplementary material for: Expression Analysis of Lipocalin 2 (LCN2) in Reproductive and Non-Reproductive Tissues of Esr1-Deficient Mice
Source: Int J Mol Sci. 2023 May 25;24(11):9280. doi: 10.3390/ijms24119280 (PMC10252910; doi:10.3390/ijms24119280)
Supplement: Supplementary file 1 [file ijms-24-09280-s001.zip › ijms-2396631-supplementary.pdf]

International Journal of
Molecular Sciences

MDPI

Supplements

Expression Analysis of Lipocalin 2 (LCN2) in Reproductive and Non-Reproductive Tissues of *Esr1*-Deficient Mice

Jan C. Kessel, Ralf Weiskirchen * and Sarah K. Schröder *

Table S1. Estrous cycle-staging based on histological features

	Histological feature	Proestrus	Estrus	Metestrus	Diestrus
**Vagina**	Mitosis	↑ - ↑↑	↑	0	0 - ↑
	Apoptosis	0	0 - ↑	↑↑	0
	Desquamation	0	0 - ↑	↑↑↑	0
	Cornified epithelia	↑ - ↑↑	↑↑ - ↑↑↑	0	0 - ↑
	Mucus layer	↑	↑ - ↑↑	0	0 - ↑
	Stratum Granulosum	↑	↑	0	0 - ↑
**Uterus**	Mitosis	↑↑ - ↑↑↑	0 - ↑	0 - ↑	↑
	Apoptosis	0 - ↑	↑↑ - ↑↑↑	↑↑↑	0 - ↑
	Epithelia	cuboidal - columnar	columnar	columnar - cuboidal	cuboidal
	Pseudostratification	↑↑ - ↑↑↑	↑↑ - ↑↑↑	↑ - ↑↑	0
	Stromal edema	↑	0 - ↑	0	0 - ↑
	Leukocyte infiltration	0 - ↑	↑↑ - ↑↑↑	↑↑ - ↑↑↑	0 - ↑
	Dilated lumen	↑↑ - ↑↑↑	↑↑ - ↑↑↑	↑ - ↑↑	0
**Ovary**	Basophilic c.l.	from previous cycles	↑↑↑	↑↑	↑
	Eosinophilic c.l.	from previous cycles	from previous cycle	↑	↑↑↑
	Degenerating c.l.	↑↑↑	↑	from previous cycle	from previous cycle
	Fibrotic tissue in c.l.	↑	0	0	↑
	Oocytes in oviduct	0	↑	0	0

Abreviations: c.l., corpus luteum; 0, no expression; ↑, low expression; ↑↑, moderate expression; ↑↑↑, strong expression.

Table S2: Antibodies used for protein analysis

Protein	Dilution	Clonality/Host	Company	Catalog No.
**Primary antibodies (Western blot)**				
GAPDH	1:1,000	mono, mouse	Santa Cruz Biotechnology, Dallas, TX, USA	sc-32233
β-actin	1:10,000	mono, mouse	Sigma-Aldrich, Taufkirchen, Germany	A5441
Vinculin	1:1,000	mono, mouse	ProteinTech® GmbH, Planegg-Martinsried, Germany	66305-1-Ig
Transferrin	1:1,000	poly, rabbit	ProteinTech® GmbH	17435-1-AP
ERα (6F11)	1:1,000	poly, mouse	Thermo Fisher Scientific, Waltham, MA, USA	MA1-27107
LCN2	1:800	poly, goat	R&D Systems, Minneapolis, MN, USA	AF3508
STAR	1:1,000	poly, rabbit	ProteinTech® GmbH	12225-1-AP
**Secondary antibodies (Western blot)**				
goat anti-rabbit IgG (H+L), HRP	1:5,000	poly, goat	Thermo Fisher Scientific	31460
goat anti-mouse IgG (H+L), HRP	1:5,000	poly, goat	Thermo Fisher Scientific	31430
mouse anti-goat IgG (H+L), HRP	1:5,000	poly, mouse	Thermo Fisher Scientific	31400
**Secondary antibodies (IF and IHC)**				
Donkey anti-goat IgG (H+L) Alexa Fluor™ 488	1:300	poly, donkey	Thermo Fisher Scientific	A-11055
Donkey anti-rabbit IgG (H+L) Alexa Fluor™ 555	1:300	poly, donkey	Thermo Fisher Scientific	A-31572
Rabbit anti-mouse IgG biotinylated	1:200	poly, rabbit	DAKO Agilent Technologies, Santa Clara, CA, USA	P026002-2
Rabbit anti-goat IgG biotinylated	1:300	poly, rabbit	DAKO Agilent Technologies	P044901-2

Abbreviations: poly, polyclonal; mono, monoclonal; IF, immunofluoresence; IHC, immunohistochemistry.

Table S3. Primers used for RT-qPCR

Mouse Gene	Accession No.	Primer (5'→3')
Actb	NM_007393.5	For: 5′-ctctagacttcgagcaggagatgg-3′<br>Rev: 5′-atgccacaggattccatacccaaga-3′
Esr1	NM_007956.5	For: 5′-tgccgtgtgcaatgactatg-3′<br>Rev: 5′-tttcatcatgcccacttcgt-3′
Il1b	NM_008361.4	For: 5′-gagctgaaagctctccacctc-3′<br>Rev: 5′-ctttcctttgaggcccaaggc-3′
Itgam	NM_001082960.1	For: 5′-caatagccagcctcagtgc-3′<br>Rev: 5′-gagcccaggggagaagtg-3′
Lcn2	NM_008491.1	For: 5′-ccatctatgagctacaagagaacaat-3′<br>Rev: 5′-tctgatccagtagcgacagc-3′
Mpo	NM_010824.2	For: 5′-gatggaatggggagaagctc-3′<br>Rev: 5′-gcaggtagtcccggtatgtg-3′
Rps6	NM_009096.3	For: 5′-cccatgaagcaaggtgttct-3′<br>Rev: 5′-acaatgcatccacgaacaga-3′
Star	NM_011485.5	For: 5′-gaggttccacctgtgtgctg-3′<br>Rev: 5′-caggtggttggcgaactcta-3′
Tnf	NM_013693.3	For: 5′-accacgctcttctgtctactga-3′<br>Rev: 5′-tccacttggtggtttgctacg-3′

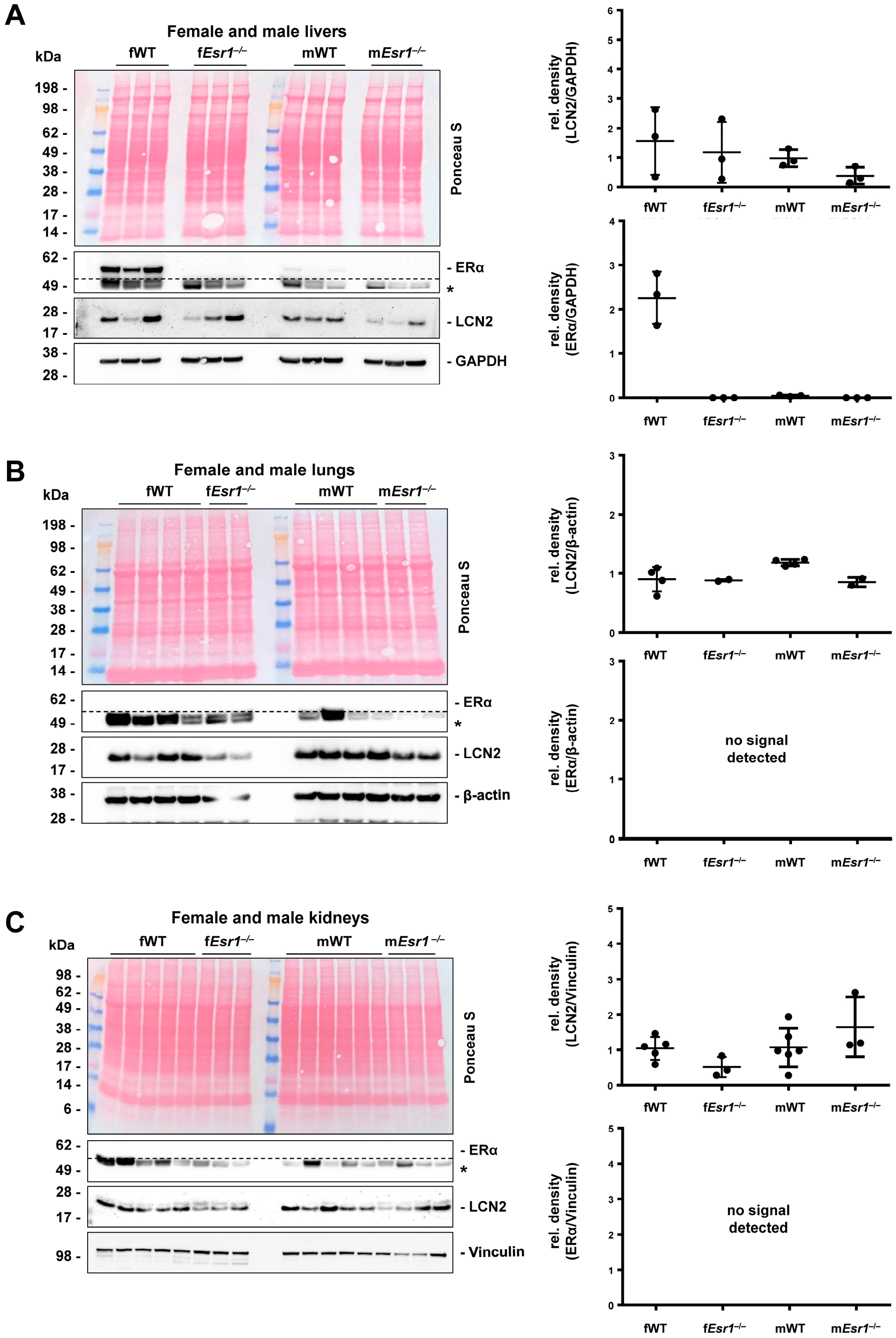

Figure S1. Representative Western blot analysis of non-reproductive organs. Tissue samples from (**A**) livers (wild type (WT): n=3, *Esr1*$^{-/-}$: n=3), (**B**) lungs (WT: n=4, *Esr1*$^{-/-}$: n=2), and (**C**) kidneys (WT: n=5, *Esr1*$^{-/-}$: n=3) of female (f) and male (m) animals were dissected and prepared for Western blot analysis and probed for expression of LCN2 and ERα. Expression of GAPDH (livers), β-actin (lungs), vinculin (kidneys), and Ponceau S stainings were used to demonstrate equal protein loading. Unspecific bands are marked with asterisks (*) and are separated from specific bands by dashed lines. Protein expression of LCN2 was quantified densitometrically and plotted relative to the expression of the loading control. Data are displayed as means ± SD.

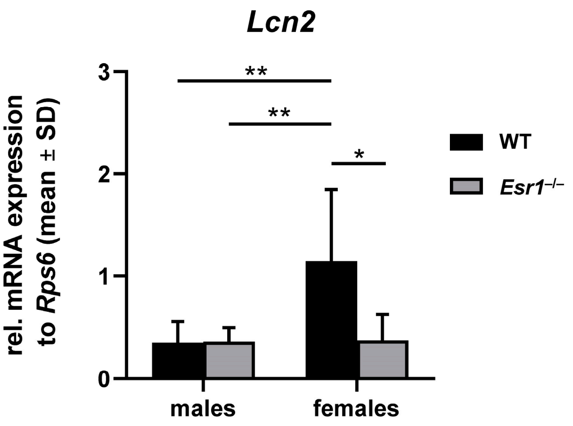

Figure S2. Hepatic *Lcn2* mRNA expression. Liver mRNA from wild type (WT) (female n=9, male n=8) and *Esr1*-knockout (female n=6, male n=8) mice were prepared and the expression of *Lcn2* was analysed by RT-qPCR. In case of normal distribution Student's *t*-test was chosen for statistical analysis, otherwise a Mann-Whitney test was performed. Significant differences between groups are marked with asterisks: * p<0.05, ** p<0.01. Data are displayed as means ± SD.

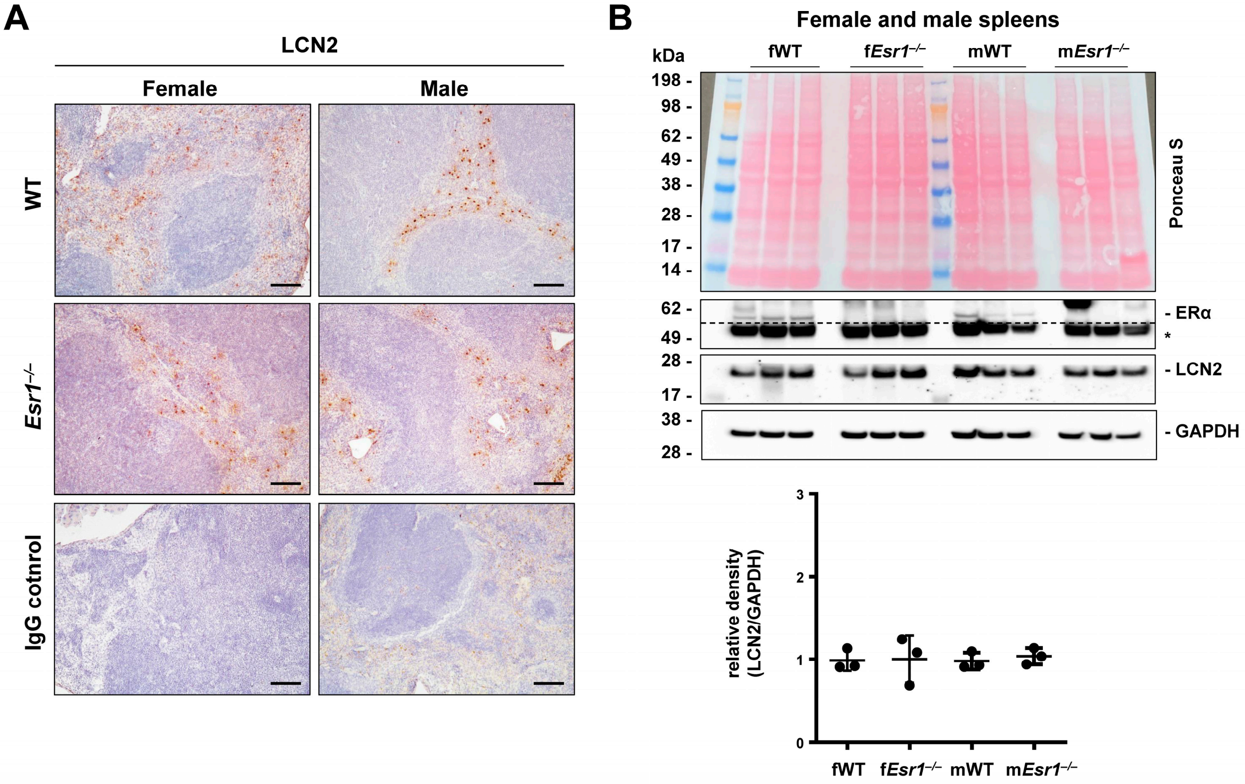

Figure S3. LCN2 expression in spleen. Spleen tissue samples from wild type (WT, female: n=3, male: n=3) and *Esr1*-knockout (female: n=3, male: n=3) mice were either embedded in paraffin for immunostaining or processed for protein analysis. (**A**) Immunohistochemical detection of LCN2 was performed in spleen tissues. Isotope-negative IgG controls were used to demonstrate antibody's specificity. Magnifications: 100x, scale bars: 100 μm. (**B**) Spleen samples were analysed by Western blot analysis for LCN2 and ERα protein expression. Expression of GAPDH and Ponceau S stain were used as a loading control. Unspecific bands are marked with asterisks (*) and are separated from specific bands by dashed lines. Protein expression of LCN2 was quantified densitometrically and plotted relative to GAPDH expression. Data are displayed as means ± SD.

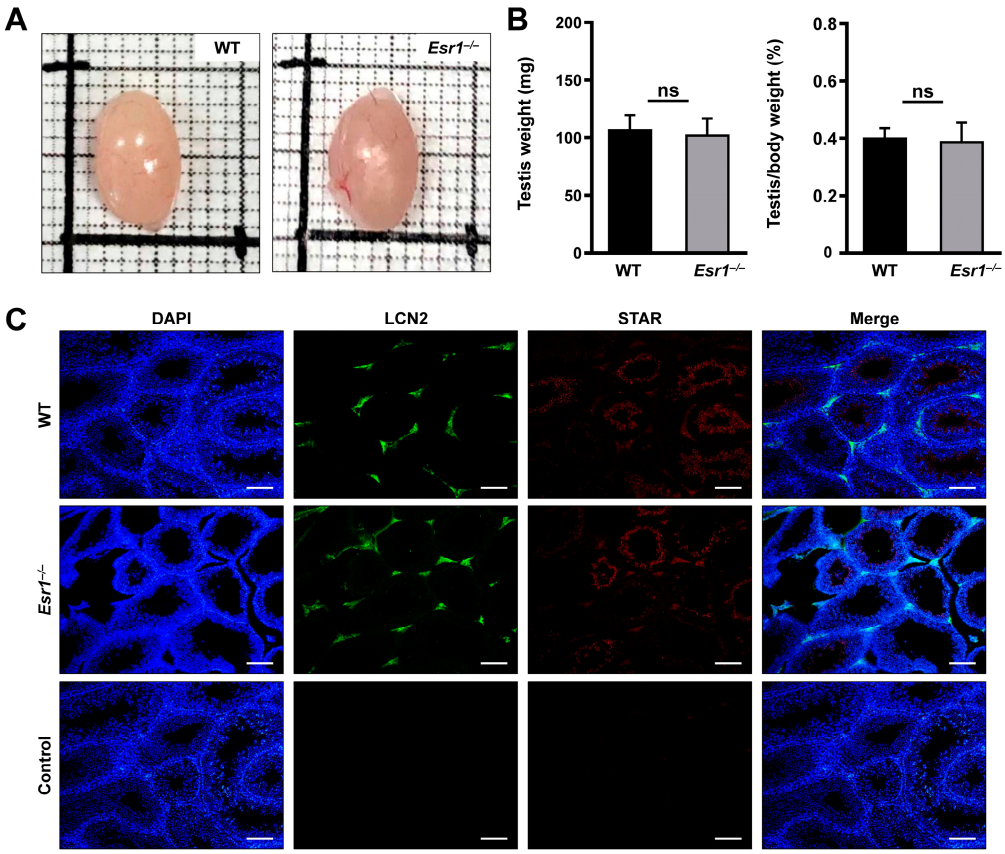

Figure S4. Testes weights of wild type (WT) and *Esr1*-depleted mice. (**A**) Testes were dissected from adult WT (n = 8) and *Esr1*$^{-/-}$ males (n = 8) and placed on scale paper to examine phenotypic appearance. (**B**) Total testes weights were measured. Testis-to-body weight ratios were calculated for each genotype and are shown in percentage. For statistical analysis, a Student's *t*-test was performed. Data are displayed as means ± SD and non-significant results are marked with ns. (**C**) Tissue samples were subjected to immunofluorescence-double-staining for LCN2 and steroidogenic acute regulatory protein (STAR) using DAPI as a counterstain. Magnifications: 100x, scale bars: 100 µm.

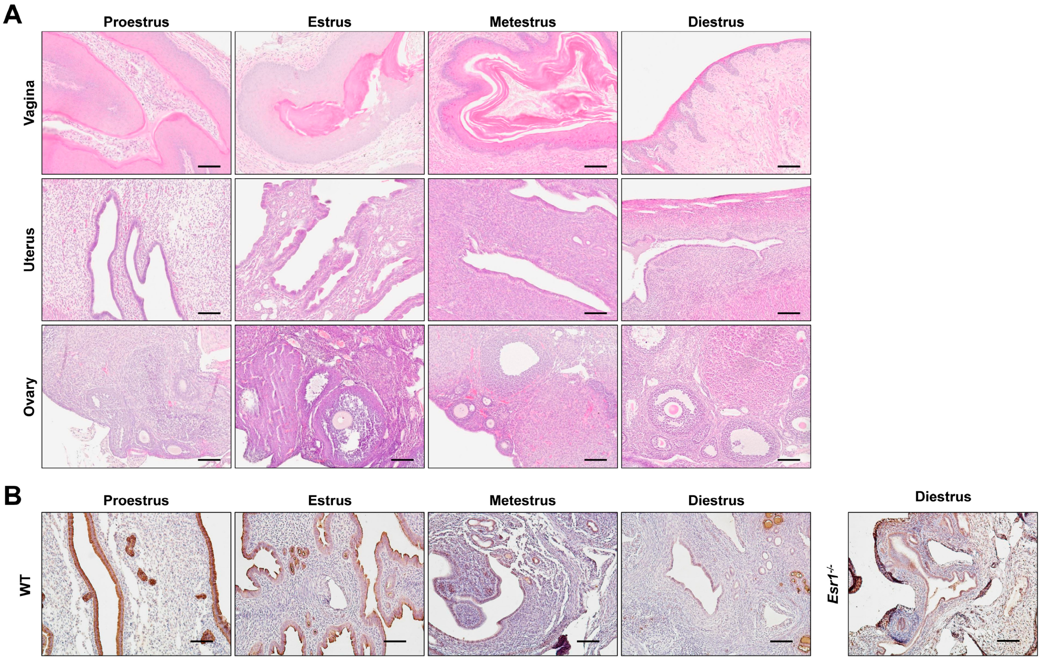

Figure S5. Determination of estrous phases as determined by histological examination. Reproductive tracts from female wild type (WT, n=8) and *Esr1*$^{-/-}$ mice (n=6) were dissected and embedded in paraffin for further analysis staining. (**A**) Histological features in major female reproductive organs (vagina, uterus, and ovary) were hematoxylin and eosin stained to determine the estrous cycle phase. Magnifications: 10x, scale bars: 100 µm. (**B**) Immunohistochemical detection of LCN2 protein expression in WT and *Esr1*$^{-/-}$ uterine tissues. Since *Esr1*-deficient animals do not develop different estrous phases but are in a constant diestrus phase, this is only shown in a single representative staining. Magnifications: 100x, scale bars: 100 µm.

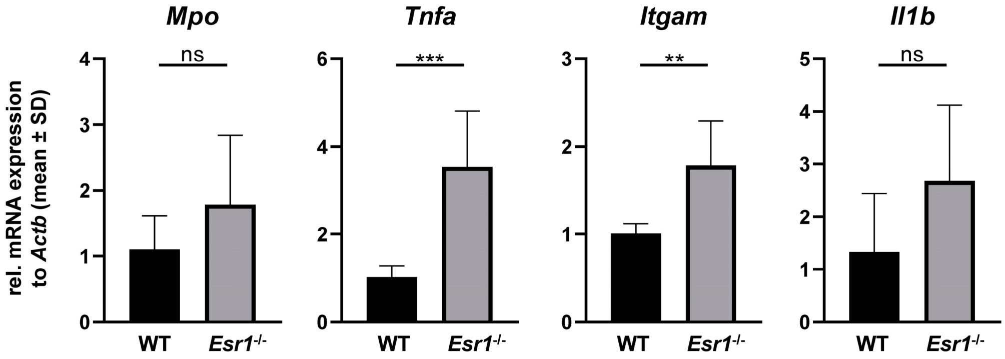

Figure S6. Detection of inflammatory markers in ovarian tissue by RT-qPCR. Ovaries from female wild type (WT, n=8) and *Esr1*-knockout mice (n=6) were dissected, prepared for RNA analysis, and the expression of several inflammatory markers analysed by RT-qPCR (myeloperoxidase, *Mpo*; interleukin-1β, *Il-1b*; tumour necrosis factor-α, *Tnfa*; integrin subunit alpha M, *Itgam*). Data are displayed as means ± SD. In case of normal distribution Student's *t*-test was chosen for statistical analysis, otherwise a Mann-Whitney test was performed. Significant differences between groups are marked by asterisks: ** p<0.01, *** p<0.001.
